# Sensitivity and specificity of two WHO approved SARS-CoV2 antigen assays in detecting patients with SARS-CoV2 infection

**DOI:** 10.1186/s12879-022-07240-6

**Published:** 2022-03-22

**Authors:** Chandima Jeewandara, Dinuka Guruge, Pradeep Darshana Pushpakumara, Deshan Madhusanka, Tibutius Thanesh Jayadas, Indika Prasad Chaturanga, Inoka Sepali Aberathna, Saubhagya Danasekara, Thilagaraj Pathmanathan, Deshni Jayathilaka, Gayasha Somathilaka, Heshan Kuruppu, Laksiri Gomes, Vitjith Gunasekara, Ruwan Wijayamuni, Graham S. Ogg, Gathsaurie Neelika Malavige

**Affiliations:** 1 Allergy Immunology and Cell Biology Unit, Department of Immunology and Molecular Medicine, Faculty of Medical Sciences, University of Sri Jayewardenepura, Nugegoda, Sri Lanka; 2 Colombo Municipality Council, Colombo, Sri Lanka; 3 Ministry of Health, Colombo, Sri Lanka; 4 MRC Human Immunology Unit, MRC Weatherall Institute of Molecular Medicine, University of Oxford, Oxford, UK

**Keywords:** SARS-CoV-2, Rapid antigen tests, PCR, Abbott Ag test, SD-Biosensor Ag test, Antibodies

## Abstract

**Background:**

SARS-CoV-2 rapid antigen (Ag) detection kits are widely used in addition to quantitative reverse transcription PCR PCR (RT-qPCR), as they are cheaper with a rapid turnaround time. As there are many concerns regarding their sensitivity and specificity, in different settings, we evaluated two WHO approved rapid Ag kits in a large cohort of Sri Lankan individuals.

**Methods:**

Paired nasopharangeal swabs were obtained from 4786 participants for validation of the SD-Biosensor rapid Ag assay and 3325 for the Abbott rapid Ag assay, in comparison to RT-qPCR. A short questionnaire was used to record symptoms at the time of testing, and blood samples were obtained from 2721 of them for detection of SARS-CoV-2 specific antibodies.

**Results:**

The overall sensitivity of the SD-Biosensor Ag kit was 36.5% and the Abbott Ag test was 50.76%. The Abbott Ag test showed specificity of 99.4% and the SD-Biosensor Ag test 97.5%. At Ct values < 25, the sensitivity was 71.3% to 76.6% for the SD-Biosensor Ag test and 77.3% to 88.9% for the Abbott Ag test. The Ct values for all genes (RdRP, S, E and N) tested with all RT-qPCR kits were significantly lower for the positive results of the Abbott Ag test compared to the SD-Biosensor test. 209 (48.04%) individuals who had antibodies gave a positive RT-qPCR result, and antibody positivity rates were higher at Ct values > 30 (46.1 to 82.9%). 32.1% of those who gave a positive result with the SD-Biosensor Ag test and 26.3% of those who gave positive results with the Abbott Ag test had SARS-CoV-2 antibodies at the time of detection.

**Conclusions:**

Both rapid Ag tests appeared to be highly sensitive in detecting individuals at lower Ct values, in a community setting in Sri Lanka, but it will be important to further establish the relationship to infectivity.

## Background

Quantitative real time PCR (RT-qPCR), with use of specific primers targeting two or three genes of the SARS-CoV-2 virus is considered as the gold-standard in the diagnosis of a patient infected with the virus [1, 2]. However, RT-qPCR test is a difficult complex test to use in community screening [3]. Due to the high cost, the need of dedicated equipment and trained individuals, use of RT-qPCR has been challenging for many developing countries with scare resources. In order to fulfill this need many diagnostic tests which are faster and cheaper, such as loop-mediated isothermal amplification (RT-LAMP) for detection of viral RNA [4], CRISPR-based assays [5] and many different types of lateral flow antigen detection assays [1, 6, 7] were developed.

The rapid antigen tests (RATs) are convenient to use due to the very fast turnaround time, their ability to test the individuals at the point of care and to their low cost. However, there have been many concerns regarding their poor sensitivity and sometimes the occurrence of false-positive results [7, 8]. While these RATs appear to be the ideal test in community surveillance programs, their pretest probability depends on the prevalence of SARS-CoV-2 in the community [7]. The pre-test probability is lower in communities with lower prevalence rates giving rise to higher false positive rates [7, 8]. The World Health Organization recommends a minimum of 80% sensitivity and 97% specificity in the RATs that are used for diagnostic purposes [8]. However, the studies that have assessed the sensitivity and specificity of the currently approved RATSs, have varied in study design and in some have been evaluated in a relatively small number of individuals [9]. Furthermore, it has been reported that certain RT-qPCR kits can give false negative results due to certain mutations that occur in the N protein [10].

Although the sensitivity of these RATs have been questioned, they are thought to be adequately sensitive to detect individuals with higher viral loads, especially during the early phases of infection and therefore, who are likely to be more infectious [9]. Therefore, the WHO has recommended their use to investigate certain outbreak situations, to monitor disease trends in communities and for early detection and isolation of infected individuals in setting where there is a high degree of community transmission [9]. Although RAT tests are not recommended to screen covid-19 in especially elective procedures such as elective surgery, blood donation, and where there is a low community prevalence [9], many countries use the RAT tests in these conditions due to the costs and time associated with the PCRs. As the performance of these RATs is known to vary in different settings, with different SARS-CoV-2 variants, we proceeded to evaluate the performance of two of the test kits, in a large cohort in individuals in the city of Colombo, Sri Lanka.

## Methods

### Participants

The Colombo Municipality Council (CMC) has a population of 752,993 individuals in an area of 37.3 km^2^ and is one of the highest population dense areas (20,187.5 persons/km^2^) in the country. The highest number of COVID-19 cases were reported in the CMC area from October 2020 to mid-January 2021 with 13,216 cases reported during this period. 8470/13216 of these individuals were recruited in this study. Therefore, in order to identify cases in the community, RT-qPCR was carried out in primary contacts of cases and also during community surveillance. Since the SD-Biosensor and Abbott RATs were first used in Sri Lanka since the end of November 2020, individuals residing in the CMC were invited to participate in this study. Paired nasopharyngeal swabs for each participant was obtained for the RAT and RT-qPCR. A short questionnaire was used to record symptoms at the time of testing, and self-reported symptoms of fever, sore throat, cough and diarrhoea were recorded. In 2721 of the participants, a blood sample was also obtained for the detection of SARS-CoV-2 antibodies, following informed written consent. Both RNA and separated serum were stored at − 80 °C.

### Informed consent and human experimentation guidelines

Informed written consent was obtained from all participants, from whom a blood sample was collected (n = 2721). Written consent was not taken when obtaining a nasopharyngeal swab, as this was carried out to identify individuals who were infected from the SARS-CoV-2 virus in the community, as a part of the Ministry of Health policy at that time. As the nasopharyngeal swabs for RT-qPCR was sent to our laboratory for routine diagnostic testing in these individuals as a part of the surveillance process of the Ministry of Health, the Ethics committee approved using this data for this study, with deidentification of individuals. Ethics approval for the study was obtained from the Ethics Review Committee of the University of Sri Jayewardenepura (01/2020).

### Realtime RT-qPCR for detection of SARS CoV-2

Nasopharyngeal swabs samples of the participants were lysed and RNA was extracted from the viral transport media using QIAmp Viral RNA Mini Kit (Qiagen, USA, Cat: 52906) and used to detect the presence of the SARS-CoV-2 virus using four different PCR kits; Allplex™ SARS-CoV-2 Assay (SeeGene, Korea), TaqPath™ COVID-19 RT-qPCR Kit (Thermo Fisher Scientific, USA), STANDARD M nCoV Real-Time Detection kit (SD Biosensor, Korea) and COVID-19 Real-time PCR Kit (HBRT-COVID-19) (Chaozhou Hybribio Biochemistry Ltd, China) as different kits were available in our laboratory during different time periods. The RT-qPCR was carried out according to the manufacturer’s instructions. As per directive of the Ministry of Health, Sri Lanka, the Ct values were indicated in the diagnostic report issued to patients and hospitals. Therefore, in order to have consistency in reporting, the cycle threshold value (Ct) of ≤ 38 for two or more genes was considered as a positive PCR result.

### SD Biosensor and Abbott RAT

Nasopharyngeal swabs samples of 4786 participants were used for validation of the SD-Biosensor RAT and 3325 for the Abbott RAT. Dedicated sterilized nasopharyngeal swabs in the kit were used to collect the sample. Samples for the Abbott PanbioTM COVID-19 RAT (Abbott, Germany) and the Standard Q covid 19 Ag test (SD Biosensor, Korea) were collected, and the assay carried out and interpreted according to the manufacturer’s instructions. All the samples were processed within an hour of the sample collection.

### Detection of antibodies to SARS-CoV-2

SARS-COV-2 specific total antibodies (IgM, IgG and IgA) were assessed in 2721 individuals using WANTAI SARS-CoV-2 Ab ELISA (Beijing Wantai Biological Pharmacy Enterprise, China). The blood samples in these individuals were obtained at the same time when paired nasopharangeal samples were taken for RT-qPCR/RAT. The Wantai ELISA assay was shown to have a sensitivity of 98% [11]. It was also found to be 100% specific when tested in over 100 serum samples obtained in year 2018, in Sri Lankan individuals [12]. The assay was carried out and results were interpreted according to manufacturers’ instructions.

### Statistical analysis

GraphPad Prism version 6 was used for statistical analysis. As the data were not normally distributed, differences in means were compared using the Mann–Whitney U test (two tailed). The descriptive statistics including the frequencies, percentages with 95% confidence intervals, cross-tabulations, measures of sensitivity, specificity, positive and negative predictive values were calculated using the MS Excel and R software tool (R version 3.3.3 (2017-03-06) and R-Studio Version 1.3.959). The formula for calculating the measures are as follows [13].

- $${\text{Sensitivity}} = \left[ {{{{\text{True}}\;{\text{Positives}}} \mathord{\left/ {\vphantom {{{\text{True}}\;{\text{Positives}}} {\left( {{\text{True}}\;{\text{Positives}} + {\text{False}}\;{\text{Negatives}}} \right)}}} \right. \kern-\nulldelimiterspace} {\left( {{\text{True}}\;{\text{Positives}} + {\text{False}}\;{\text{Negatives}}} \right)}}} \right] \times 100.$$
- $${\text{Specificity}} = \left[ {{{{\text{True}}\;{\text{Negatives}}} \mathord{\left/ {\vphantom {{{\text{True}}\;{\text{Negatives}}} {\left( {{\text{False}}\;{\text{Positives}} + {\text{True}}\;{\text{Negatives}}} \right)}}} \right. \kern-\nulldelimiterspace} {\left( {{\text{False}}\;{\text{Positives}} + {\text{True}}\;{\text{Negatives}}} \right)}}} \right] \times 100.$$
- $${\text{Positive}}\;{\text{predictive}}\;{\text{value}}\left( {{\text{PPV}}} \right) = \left[ {{{{\text{True}}\;{\text{Positives}}} \mathord{\left/ {\vphantom {{{\text{True}}\;{\text{Positives}}} {\left( {{\text{True}}\;{\text{Positives}} + {\text{False}}\;{\text{Positives}}} \right)}}} \right. \kern-\nulldelimiterspace} {\left( {{\text{True}}\;{\text{Positives}} + {\text{False}}\;{\text{Positives}}} \right)}}} \right] \times 100.$$
- $${\text{Negative}}\;{\text{predictive}}\;{\text{value}}\left( {{\text{NPV}}} \right) = \left[ {{{{\text{True}}\;{\text{Negatives}}} \mathord{\left/ {\vphantom {{{\text{True}}\;{\text{Negatives}}} {\left( {{\text{False}}\;{\text{Negatives}} + {\text{True}}\;{\text{Negatives}}} \right)}}} \right. \kern-\nulldelimiterspace} {\left( {{\text{False}}\;{\text{Negatives}} + {\text{True}}\;{\text{Negatives}}} \right)}}} \right] \times 100.$$

## Results

### Performance of the SD-Biosensor RAT

In order to evaluate the sensitivity and specificity of the SD-Biosensor RAT, paired samples were tested in 4786 individuals. The sensitivity and specificity of the SD-Biosensor RAT was evaluated against three PCR kits; SeeGene (n = 2427), Taqpath (n = 1667) and SD Biosensor (n = 692) as different kits were available in our laboratory during different time periods. These calculations were carried out after excluding the samples which gave a negative result in the internal control (those that had inadequate RNA) in the RT-qPCR assay (n = 59). All these three kits have the RdRP gene of the SARS-CoV2 as a target, while SeeGene has N and E genes as the other target genes, Taqpath has N and S as the other genes and SD-Biosensor E gene as the other target gene.

Of the 4786 individuals, 893 gave a positive RT-qPCR result, 3849 were negative and the remaing gave an inconclusive result. Of the 874 RT-qPCR positive individuals, the RAT was positive in 316 (36.2%) individuals, negative in 556 (63.6%) and gave an inconclusive result 2 individuals. Therefore, the overall sensitivity of the SD-Biosensor RAT was 36.24% (95% CI − 33.1% to 39.5%). Of those who had a negative RT-qPCR result, 94 (2.4%) gave a positive RAT result and therefore, the specificity was 97.6% (95% CI 97% to 98%). The positive predictive value (PPV) of the assay compared to RT-qPCR was 77% and the negative predictive value (NPV), was 87.1%. The sensitivity of this RAT at different Ct values for different PCR kits is shown in Table 1.

**Table 1:** Positivity rate of the SD-Biosensor rapid Ag kit for different RT-qPCR kits at different Ct values

PCR Kit (number of PCR positive samples)	Avg Ct value for PCR	Number of samples used	Sensitivity of the SD-Biosensor Ag kit (%)	95% CI lower limit (%)	95% CI upper limit (%)
SeeGene Kit (n = 505)	< 25	177	76.6	69.5	82.5
	26–30	130	36.2	28.1	45.1
	31–38	198	6.1	3.3	10.6
TAQPATH Kit (n = 186)	< 25	87	71.3	60.4	80.2
	26–30	77	13	6.7	23
	31–38	22	9.5	1.7	31.8
SD-Biosensor RT-qPCR Kit (n = 183)	< 25	61	72.1	59%	82.5
	26–30	51	7.8	2.5%	19.7
	31–38	71	0%	0	6.4

**Fig. 1:**
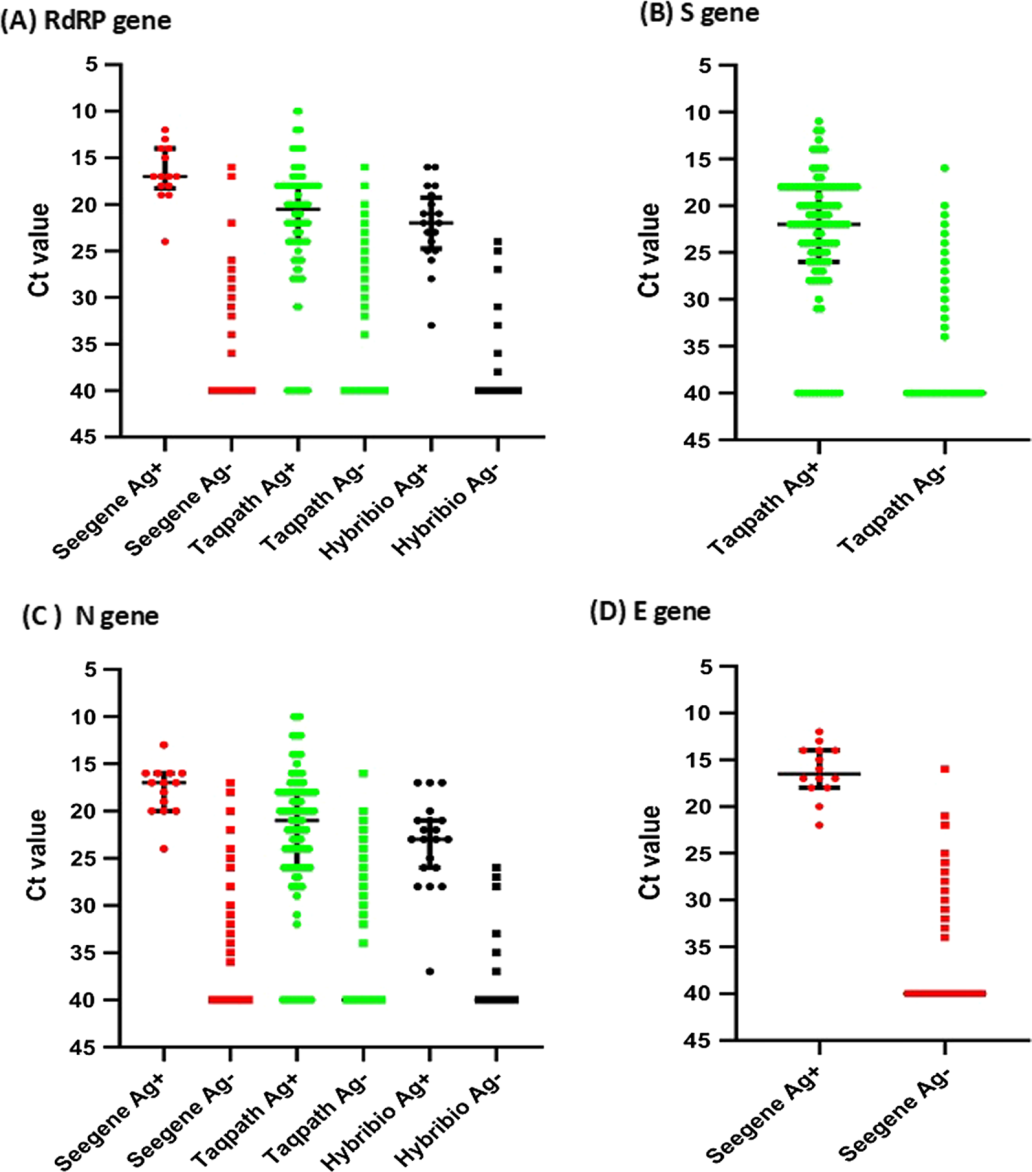
Cycle threshold (Ct) values for different gene targets in different RT-qPCR kits compared to the results of the SD-Biosensor rapid Ag kit. The Ct values for the RdRP gene (**A**), S gene (**B**), N gene (**C**) and E gene (**D**) was compared for different kits for Ag positive and Ag negative samples. The lines indicate the median and the error bars the IQR

The viral load in a respiratory sample inversely correlates with the cycle threshold (Ct). As individuals with lower Ct values, have higher viral loads and likely to be more infectious [14], we evaluated the sensitivity of this RAT at different Ct values of the RdRp gene, E gene, N gene and S that was used in the RT-qPCR assay (Fig. 1). For the RdRp gene, the median Ct value of SD-Biosensor RAT positive samples was 26 (IQR 23 to 40) by the SeeGene kit, 25 (IQR 19 to 40) by the Taqpath kit and 10 (IQR 10 to 23) by the SD-Biosensor RT-qPCR kit (Fig. 1A). For the RAT negative samples, the median Ct values for all three kits was 40 (IQR 40 to 40). The S gene target was only present in the Taqpath kit and the median Ct values for RAT positive samples was 26 (IQR 19.75 to 40) (Fig. 1B). Two PCR kits had primers targeting the N gene and the median Ct value of SD-Biosensor RAT positive samples was 17 (IQR 16 to 20) for the SeeGene kit and 26 (IQR 22 to 39) by the Taqpath kit and 26 (IQR 20 to 40) (Fig. 1C). Two kits had primers targeting the E gene and the median Ct value of SD-Biosensor Ag positive samples in the SeeGene kit was 24 (IQR 19 to 35) and for the SD-Biosensor RT-qPCR kit it was 19 (IQR11 to 20) (Fig. 1D).

### Performance of the Abbot RAT

For determining the sensitivity and specificity of the Abbott SARS-CoV2 RAT, the RAT and RT-qPCR were carried out in 3325 individuals. The sensitivity and specificity of the Abbott RAT were evaluated against three PCR kits; SeeGene (n = 555), Taqpath (n = 2490) and Hyrbribio (n = 280), as different kits were available in our laboratory during different time periods. Of the 3325 individuals, 327 gave a positive RT-qPCR result, 3288 were negative and 10 gave an inconclusive result. Of the 287(8.63%) RT-qPCR positive individuals, the RAT was positive in 151 (52.6%) individuals and negative in 136 (47.4%). Of those who had a negative RT-qPCR result, 13 (0.43%) gave a positive RAT result. Therefore, the overall sensitivity of the RAT was 52.6% (95% CI 46.7% to 58.5%) and the specificity was 99.6% (95% CI 99.2% to 99.8%). The PPV of the assay compared to RT-qPCR was 92.1% and the NPV was 95.7%. The sensitivity of this RAT at different Ct values for different PCR kits is shown in Table 2.

**Table 2:** Positivity rate of the Abbott rapid Ag kit for different RT-qPCR kits at different Ct value

PCR Kit (number of PCR positive samples)	Avg Ct value for PCR	Number of samples used	Sensitivity of the Abbott Ag kit (%)	95% CI lower limit (%)	95% CI upper limit (%)
SeeGene Kit (n = 53)	< 25	22	77.3	54.2	91.3
	26–30	14	7.1	0.4	35.8
	31–38	17	0	0	22.9
TAQPATH Kit (n = 201)	< 25	118	81.4	72.9	87.7
	26–30	75	18.7	10.9	29.7
	31–38	8	25	4.5	64.4
Hybri-Bio Kit (n = 32)	< 25	18	88.9	63.9	98.1
	26–30	5	60	17	92.7
	31–38	9	11.1	0.6	49

**Fig. 2:**
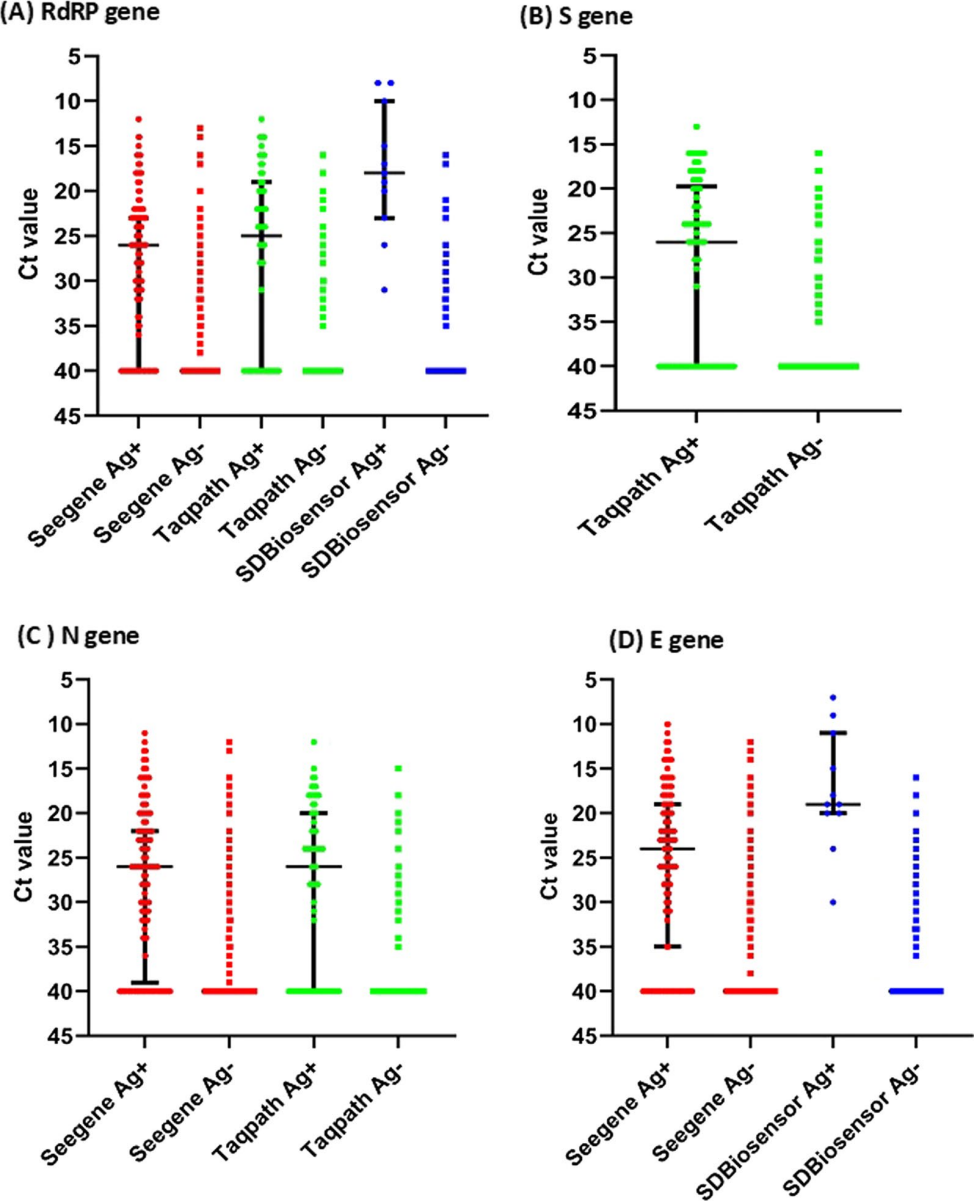
Cycle threshold (Ct) values for different gene targets in different RT-qPCR kits compared to the results of the Abbott rapid Ag kit. The Ct values for the RdRP gene (**A**), S gene (**B**), N gene (**C**) and E gene (**D**) was compared for different kits for Ag positive and Ag negative samples. The lines indicate the median and the error bars the IQR

The sensitivity and specificity of this RAT was also evaluated for different Ct values of the RdRp gene, E gene, N gene and S that was used in the RT-qPCR assay (Fig. 2). For the RdRp gene, the median Ct value of Abbott RAT positive samples was 17 (IQR 14 to 18.25) for the SeeGene kit, 20.5 (IQR 18 to 24) by the Taqpath kit and 22 (IQR 19.25 to 24.75) by the Hybribio kit (Fig. 2A). For the RAT negative samples, the median Ct values for all three kits was 40 (IQR 40 to 40).

For the S gene, which was only targeted by the Taqpath kit, the median Ct value of RAT positive samples was 22 (IQR 18 to 26) (Fig. 2B).

All three PCR kits had primers targeting the N gene and the median Ct value of Abbott RAT positive samples was 17 (IQR 16 to 20) for the SeeGene kit, 21 (IQR 18 to 26) by the Taqpath kit and 23 (IQR 21 to 26) by the Hybribio kit (Fig. 2C). The SeeGene kit only had primers targeting the E gene and the median Ct value of RAT positive samples was 16.5 (IQR 14 to 18) (Fig. 2D).

### Comparison of the Ct values in the samples which gave a positive result by the SD-Biosensor vs the Abbot RAT

**Fig. 3:**
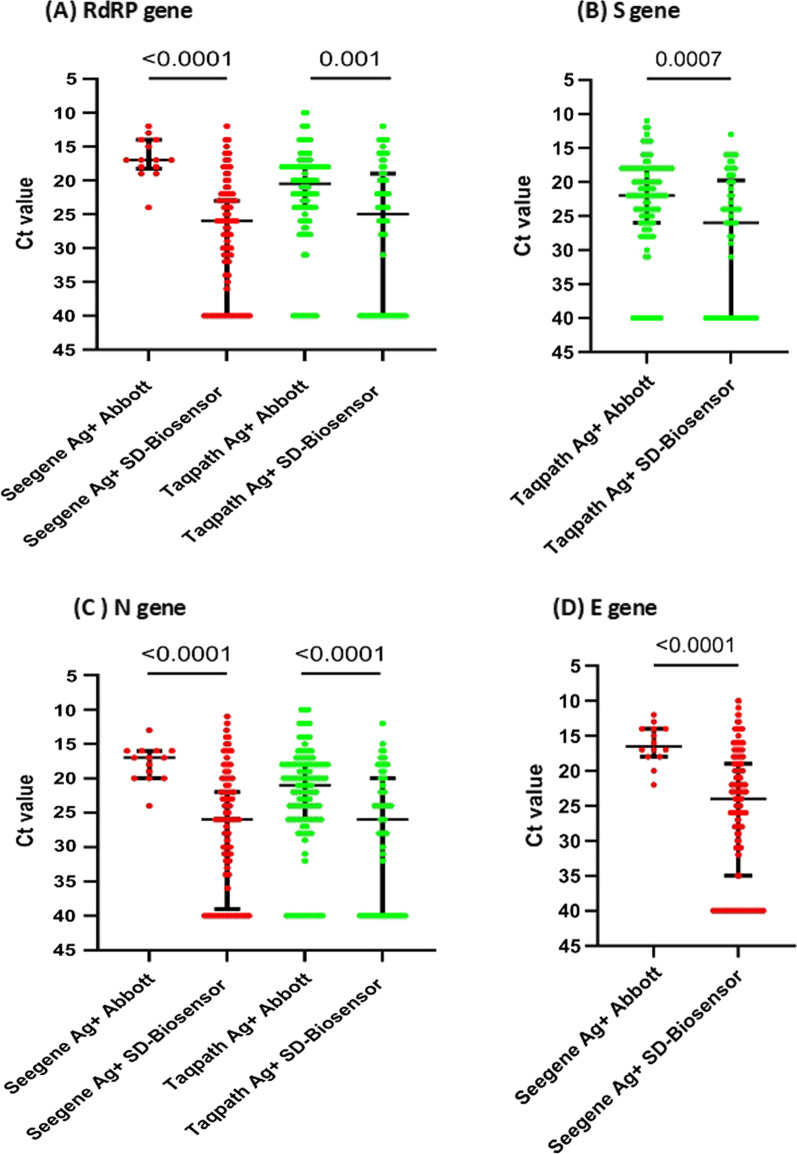
Comparison of the Ct values for different gene targets of RT-qPCR kits of positive samples from the SD-Biosensor Ag kit and the Abbott Ag kit. The Ct values for the RdRP gene (**A**), S gene (**B**), N gene (**C**) and E gene (**D**) was compared for different kits for SD-Biosensor Ag positive and Abbot Ag positive samples. The lines indicate the median and the error bars the IQR

In order to determine the differences in detection by the two RATs at different Ct values, we compared the Ct values of the samples which gave a positive result for the SD-Biosensor RAT and the Abbott RAT for the RdRp, S, N and the E genes. This comparison was only carried out for the SeeGene and the Taqpath kits as the SD-Biosensor RT-qPCR kits were not used to validate the Abbott RAT and the Hybribio PCR kit was not used to validate the SD-Biosensor RAT. For RdRP, S, N and the E gene, the Ct values of the Abbott RAT positive samples was significantly less for the SD-Biosensor RAT positive samples for both PCR kits (Fig. 3).

For the RdRP gene the Ct values of the Abbot RAT positive samples was significantly less for the SeeGene kit (p < 0.0001) than the Taqpath kit (p = 0.001) (Fig. 3A). The Ct values of the Abbott RAT positives were again significantly lower for the S gene with the Taqpath kit (p = 0.0007) (Fig. 3B) and also for the N gene in the SeeGene kit (p < 0.0001) and the Taqpath kit (p < 0.0001) (Fig. 3C).

The Ct values of the Abbott RAT positives were again significantly lower for the S gene with the Taqpath kit (p = 0.0007) (Fig. 3B) and also for the N gene in the SeeGene kit (p < 0.0001) and the Taqpath kit (p < 0.0001) (Fig. 3C). Only the Seegene kit targeted the E gene and again the Ct values of the Abbott RAT positives were again significantly lower in the Abbott RAT positive samples compared to the SD-Biosensor Ag positive samples (p < 0.0001) (Fig. 3D).

### Presence of SARS-CoV2 antibodies and the performance of the RATs

Individuals infected with the SARS-CoV-2 virus may shed virus for a prolonged period [15, 16], but are often thought to shed non-infectious, or non-culturable virus [17, 18]. The appearance of neutralizing antibodies are thought to associate with the presence of non-culturable and therefore, virus particles that are non-infectious [17]. Therefore, it has been proposed that seroconversion and Ct values of > 24 could be used to release individuals from isolation/quarantine [17]. As one of the most important uses of the SARS-CoV2 RATs it to detect people who are infectious, we proceeded to investigate their performance in individuals from the community and in those who are primary and secondary contacts of the index patient. We obtained blood samples from 2721 individuals at the same time as paired nasopharyngeal samples were obtained for RT-qPCR and antigen testing. The presence of SARS-CoV2 specific antibodies were detected using the Wantai ELISA, which detects the presence of virus specific IgA, IgM and IgG antibodies and was shown to have a sensitivity of 98% [11]. The specificity of this assay was evaluated in the Sri Lankan population in 81 serum samples obtained in 2018 and was found to be 100% specific.

Of the study participants, 435 (15.98%), were found to have SARS-CoV-2 specific antibodies, although they were not aware of any recent or past COVID-19 illness. 209 (48.04%) individuals who had antibodies gave a positive RT-qPCR result, while 226 (52.4%) SARS-CoV2 antibody positive individuals were negative for RT-qPCR. The presence of SARS-CoV2 antibodies for the three different PCR kits, at different Ct values is shown in Table 3.

**Table 3:** The presence of SARS-CoV2 specific antibodies for different RT-qPCR kits and different Ct values

Avg Ct values	Seegene	TAQPATH	SD-Biosensor
< 25	21/80 = 26.25%	15/63 = 23.81%	10/24 = 41.67%
26–30	41/52 = 78.85%	22/62 = 35.48%	12/17 = 70.59%
31–38	58/70 = 82.86%	6/12 = 50%	18/39 = 46.15%

Of those who had a positive result with the SD-Biosensor RAT 60/187 (32.1%) had detectable SARS-CoV-2 antibodies, while antibodies were detected in 432/1751 (24.6%) of those who tested negative result (Table 4). Of those who tested positive with the Abbott RAT 10/38 (26.3%) were also positive for antibodies, and 109/602 (18.1%) who tested negative for the Abbott RAT, had SARS-CoV2 specific antibodies.

**Table 4:** The presence of SARS-CoV2 specific antibodies, in those who were tested positive vs. negative for the SARS-CoV2 rapid antigen kits

	Ab Positive	Ab Negative	Total
SD-Biosensor Ag Positive	60 (32.1%)	127	187
SD-Biosensor Ag Negative	432 (24.6%)	1319	1751
Abbott Ag Positive	10 (26.3%)	28	38
Abbott Ag Negative	109 (18.1%)	493	602

### Disease symptoms and sensitivity of RAT compared to RT-qPCRs

Four symptoms were recorded at the time of testing in all participants and if they reported at least one of the four symptoms (fever, sore throat, cough or diarrhea) they were considered to be symptomatic. Of the 8470 individuals, 249 (2.94%) were symptomatic and 8221 (97.1%) were asymptomatic at the time of testing. Of those who were asymptomatic 1115 (13.2%) were PCR positive. Of those who were PCR positive, 847 (75.9%) were positive by the SD-Biosensor and 268 (24.03%) by the Abbott RAT. Of those who were asymptomatic and PCR negative, 88/7106 (1.23%) tested positive for the SD RAT and 20/7106 (0.28%) tested positive for the Abbott RAT.

209 (48.4%) of the PCR positive individuals had SARS-CoV-2 antibodies at the time of testing. 8 of them were symptomatic while 201 (96.2%) were asymptomatic. While numbers of RT-qPCR positive, antibody-positive symptomatic individuals is small as expected, there was no significant difference in RT-qPCR positivity and presence or absence of symptoms or antibody positivity.

## Discussion

In this study we have evaluated the performance of two WHO approved RATs in a large cohort of individuals (n = 8111) in an area of high prevalence (1.76%) when the dominant circulating variant was a virus of the SARS-CoV-2 B.1.411 lineage [19]. In Sri Lanka, the control strategy consisted of identifying every possible infected individual through testing of all contacts of cases and also through community screening in locations where increased numbers of SARS-CoV-2 infected individuals were identified. Therefore, it was important to evaluate if RAT were a good tool to identify infected individuals, in the community, in comparison to RT-qPCR. Compared to RT-qPCR, the overall sensitivity of the SD-Biosensor RAT was 36.2% and the Abbott RAT was 52.6%. The Abbott RAT was also found to be more specific (99.6%) than the SD-Biosensor RAT (97.6%). Therefore, in an area of a high level of transmission, 115/4786 (2.4%) individuals who were identified as being negative by RT-qPCR were given a positive result by the SD-Biosensor RAT, whereas the false positivity rates of the Abbott RAT were lower (13/3325 = 0.4%). The PPV and the NPV were also significantly better in the Abbott RAT (92.1% and 95.7%) compared to the SD-Biosensor RAT (77% and 87.1%). The Abbott RAT indicated positives at significantly lower Ct values for all genes for all the RT-qPCR kits tested compared to the SD-Biosensor RAT. As the Abbott RAT gives a positive result at lower Ct values, it is less likely to give a false positive result and therefore, has a high specificity, PPV and an NPV. The WHO has that RATs should have minimum requirement of a sensitivity of 80% and a specificity of 97% [20]. Although both kits had the required specificity, the SD-Biosensor RAT had lower sensitivity than 80%, at Ct values < 25, with all the RT-qPCRs that were compared in this study.

Studies carried out in hospital settings, have reported higher sensitivity for the RAT than reported by us [21, 22]. For instance, a study carried out in hospital setting in Italy has shown higher PPV and NPV values for RAT than reported by us, with higher sensitivity rates [21]. Another study in a Swiss University hospital also reported a sensitivity of 65.3% for the SD-Biosensor RAT, which was higher than the 36.5% sensitivity reported by us [22]. RAT was shown to be more sensitive in detecting infections in symptomatic individuals compared to asymptomatic individuals [23]. Since the majority of individuals in our study had asymptomatic infection, it is possible that lower sensitivity rates reported by us compared to sensitivity rates reported in a hospital-based study in Italy, could be due to this.

Although patients who are infected with the SARS-CoV-2 virus have the highest viral loads during early illness, which associates with infectivity, they can shed non-infectious virus for many weeks [24, 25]. Lower Ct values (< 20) have shown to associate with infectivity [26, 27] and also associate with a higher probability of culturing the virus [28]. Therefore, the RATs that detect patients having higher viral loads (lower Ct values), are thought to be ideal in identifying individuals who are most infectious and likely to spread the virus. Indeed, the Abbot RAT gave positive results for samples with a median Ct value of 17 to 23 for different genes and different PCR kits, while the SD RAT gave positive results for samples with a median Ct value between 18 and 26 for different gene targets and kits. Therefore, these RAT do appear to be useful in identifying patients with higher viral loads, who are possibly more infectious.

It has been established that individuals with mild or asymptomatic infection are unlikely to be infectious 7 to 10 days since the onset of symptoms [18, 29]. SARS-CoV-2 specific antibodies become detectable after 7 days since onset of illness and sometimes much later in patients with asymptomatic of mild illness [13, 15]. In our cohort of patients, 97.1% were tested positive were asymptomatic at the time of testing. 48.04% of these individuals had antibodies, suggesting that they had been harboring the infection for some time. Therefore, a large proportion of asymptomatic individuals who are tested positive by RT-qPCR and thus isolated from the community seem likely to have passed their infective period. Interestingly, of those who had Ct values of < 25, 23.8% to 41.67% had detectable antibodies. This suggests that even those with lower Ct values at time of detection are likely to have had the infection for a while and shedding non-infectious virus particles. Therefore, low Ct values per se at the time of detection in asymptomatic individuals, does not seem to indicate that the person is in the early phase of the illness. It is likely that these individuals were RAT/RT-qPCR positive with low Ct values with detectable antibodies due to recent infection, as intermittent shedding of SARS-CoV-2, has shown to occur even in asymptomatic individuals for over 2 months [15]. Furthermore, only very few cases were reported in the community in Sri Lanka (specially in this study area) in April 2020 to May 2020 [30]. Since no cases of COVID-19 was detected in the community in Sri Lanka from May 2020 to late September 2020, it is unlikely that a large number of individuals In this cohort, antibody positivity was higher with Ct values > 30, with the antibody positivity ranging from 82.86% for the SeeGene kit to 46.15% for the SD-Biosensor PCR assay. 32.1% of those who gave a positive result with the SD-Biosensor RAT and 26.3% of those who were positive with the Abbott RAT, had SARS-CoV-2 antibodies at the time of testing. Therefore, RAT do not appear to necessarily detect only infectious individuals, as a significant proportion of those who tested positive by RATs seem to have had the infection for at least more than 7 days.

One of the main limitations of this study is that the performance of these RATs was evaluated in identification of infection of primary and secondary contacts of cases and in community surveillance. As the majority of this cohort of individuals were asymptomatic when the predominant SARS-CoV-2 variant was B.1.411 [19], these RATs could give different sensitivity and specificity in individuals who are symptomatic, in a hospital setting. Furthermore, if these assays were evaluated in a location of high prevalence and again the sensitivity and specificity, PPV and NPV could be different in areas where there is a low prevalence of SARS-CoV-2 infection.

## Conclusion

Both SD-Biosensor RAT and Abbot RAT kits demonstrated a high sensitivity and specificity in identifying individuals who were acutely infected with the SARS-CoV-2 virus with Ct values < 25. Since Ct values < 25 have associated with higher viral loads and therefore, higher infectivity, they are important tools in identifying infectious individuals in the community. In addition, as these tests can be carried out at the point of care, they can be used in community screening programs and at point of entry and exit to rapidly identify possible infectious individuals.

## Data Availability

The datasets used and/or analysed during the current study are available from the corresponding author on reasonable request.

## Abbreviations

Ab: Antibody
Ag: Antigen
CMC: Colombo Municipality Council
Ct: Cycle threshold
NPV: Negative predictive value
PCR: Polymerase chain reaction
PPV: Positive predictive value
RAT: Rapid antigen test
RdRp: RNA-dependent RNA polymerase
RT-qPCR: Quantitative reverse transcription PCR

## References

[1] Tang YW, Schmitz JE, Persing DH, Stratton CW (2020). Laboratory diagnosis of COVID-19: current issues and challenges. J Clin Microbiol.

[2] Kevadiya BD, Machhi J, Herskovitz J, Oleynikov MD, Blomberg WR, Bajwa N, Soni D, Das S, Hasan M, Patel M (2021). Diagnostics for SARS-CoV-2 infections. Nat Mater.

[3] Mina MJ, Peto TE, Garcia-Finana M, Semple MG, Buchan IE (2021). Clarifying the evidence on SARS-CoV-2 antigen rapid tests in public health responses to COVID-19. Lancet.

[4] Yoshikawa R, Abe H, Igasaki Y, Negishi S, Goto H, Yasuda J (2020). Development and evaluation of a rapid and simple diagnostic assay for COVID-19 based on loop-mediated isothermal amplification. PLoS Negl Trop Dis.

[5] Broughton JP, Deng X, Yu G, Fasching CL, Servellita V, Singh J, Miao X, Streithorst JA, Granados A, Sotomayor-Gonzalez A (2020). CRISPR-Cas12-based detection of SARS-CoV-2. Nat Biotechnol.

[6] Chaimayo C, Kaewnaphan B, Tanlieng N, Athipanyasilp N, Sirijatuphat R, Chayakulkeeree M, Angkasekwinai N, Sutthent R, Puangpunngam N, Tharmviboonsri T (2020). Rapid SARS-CoV-2 antigen detection assay in comparison with real-time RT-PCR assay for laboratory diagnosis of COVID-19 in Thailand. Virol J.

[7] Centre for Disease Control U. Interim guidance for antigen testing for SARS-CoV-2. In: National center for immunization and respiratory diseases (NCIRD), Division of Viral Diseases; 2020.

[8] Peeling RW, Olliaro PL, Boeras DI, Fongwen N (2021). Scaling up COVID-19 rapid antigen tests: promises and challenges. Lancet Infect Dis.

[9] WHO. Antigen-detection in the diagnosis of SARS-CoV-2 infection using rapid immunoassays. In: Interim guidance*.* Emergencies Preparedness, WHO Headquarters (HQ); 2020. p. 9.

[10] FDA. Genetic variants of SARS-CoV-2 may lead to false negative results with molecular tests for detection of SARS-CoV-2—letter to Clinical Laboratory Staff and Health Care Providers. FDA; 2021.

[11] Weidner L, Gansdorfer S, Unterweger S, Weseslindtner L, Drexler C, Farcet M, Witt V, Schistal E, Schlenke P, Kreil TR (2020). Quantification of SARS-CoV-2 antibodies with eight commercially available immunoassays. J Clin Virol.

[12] Jeewandara C, Guruge D, Abyrathna IS, Danasekara S, Gunasekera B, Pushpakumara PD, Madhusanka D, Jayathilaka D, Ranasinghe T, Somathilaka G (2021). Seroprevalence of SARS-CoV-2 infection in the Colombo Municipality region, Sri Lanka. medRxiv.

[13] Watson J, Richter A, Deeks J (2020). Testing for SARS-CoV-2 antibodies. BMJ.

[14] Singanayagam A, Patel M, Charlett A, Lopez Bernal J, Saliba V, Ellis J, Ladhani S, Zambon M, Gopal R (2020). Duration of infectiousness and correlation with RT-PCR cycle threshold values in cases of COVID-19, England, January to May 2020. Euro Surveill.

[15] Jeewandara C, Jayathilaka D, Gomes L, Wijewickrama A, Narangoda E, Idampitiya D, Guruge D, Wijayamuni R, Manilgama S, Ogg GS (2021). SARS-CoV-2 neutralizing antibodies in patients with varying severity of acute COVID-19 illness. Sci Rep.

[16] Cevik M, Tate M, Lloyd O, Maraolo AE, Schafers J, Ho A (2021). SARS-CoV-2, SARS-CoV, and MERS-CoV viral load dynamics, duration of viral shedding, and infectiousness: a systematic review and meta-analysis. Lancet Microbe.

[17] van Kampen JJA, van de Vijver D, Fraaij PLA, Haagmans BL, Lamers MM, Okba N, van den Akker JPC, Endeman H, Gommers D, Cornelissen JJ (2021). Duration and key determinants of infectious virus shedding in hospitalized patients with coronavirus disease-2019 (COVID-19). Nat Commun.

[18] Perera R, Tso E, Tsang OTY, Tsang DNC, Fung K, Leung YWY, Chin AWH, Chu DKW, Cheng SMS, Poon LLM (2020). SARS-CoV-2 virus culture and subgenomic RNA for respiratory specimens from patients with mild coronavirus disease. Emerg Infect Dis.

[19] Jeewandara C, Jayathilaka D, Ranasinghe D, Hsu NS, Ariyaratne D, Jayadas TT, Madushanka D, Lindsey BB, Gomes L, Parker MD (2021). Genomic and epidemiological analysis of SARS-CoV-2 viruses in Sri Lanka. medRxiv.

[20] WHO. Antigen-detection in the diagnosis of SARS-CoV-2 infection. Emergencies Preparedness, WHO Headquarters (HQ); 2021. p. 20.

[21] Treggiari D, Piubelli C, Caldrer S, Mistretta M, Ragusa A, Orza P, Pajola B, Piccoli D, Conti A, Lorenzi C (2021). SARS-CoV-2 rapid antigen test in comparison to RT-PCR targeting different genes: a real-life evaluation among unselected patients in a regional hospital of Italy. J Med Virol.

[22] Jegerlehner S, Suter-Riniker F, Jent P, Bittel P, Nagler M (2021). Diagnostic accuracy of a SARS-CoV-2 rapid antigen test in real-life clinical settings. Int J Infect Dis.

[23] Parvu V, Gary DS, Mann J, Lin YC, Mills D, Cooper L, Andrews JC, Manabe YC, Pekosz A, Cooper CK (2021). Factors that influence the reported sensitivity of rapid antigen testing for SARS-CoV-2. Front Microbiol.

[24] Wolfel R, Corman VM, Guggemos W, Seilmaier M, Zange S, Muller MA, Niemeyer D, Jones TC, Vollmar P, Rothe C (2020). Virological assessment of hospitalized patients with COVID-2019. Nature.

[25] Widders A, Broom A, Broom J (2020). SARS-CoV-2: the viral shedding vs infectivity dilemma. Infect Dis Health.

[26] Coyle PV, Molawi NHA, Kacem MABH, Kahlout RAE, Kuwari EA, Khal AA, Gilliani I, Jeremijenko A, Saeb H, Thani SMA (2021). Inclusion of cycle threshold (CT) values when reporting SARS-CoV-2 RT-PCR results improves clinical Interpretation in suspected and confirmed COVID-19. medRxiv.

[27] Kampf G, Lemmen S, Suchomel M (2020). Ct values and infectivity of SARS-CoV-2 on surfaces. Lancet Infect Dis.

[28] Rao SN, Manissero D, Steele VR, Pareja J (2020). A systematic review of the clinical utility of cycle threshold values in the context of COVID-19. Infect Dis Ther.

[29] Walsh KA, Spillane S, Comber L, Cardwell K, Harrington P, Connell J, Teljeur C, Broderick N, de Gascun CF, Smith SM (2020). The duration of infectiousness of individuals infected with SARS-CoV-2. J Infect.

[30] Jeewandara C, Guruge D, Jayathilaka D, Deshan Madhusanka PA, Pushpakumara PD, Tanussiya Ramu S, Sepali Aberathna I, Saubhagya Rasikangani Danasekara DR, Pathmanathan T, Gunatilaka B (2021). Transmission dynamics, clinical characteristics and sero-surveillance in the COVID-19 outbreak in a population dense area of Colombo Sri Lanka April–May 2020. PLoS ONE.

